# Growth and Structural Characterization of *h*-LuMnO_3_ Thin Films Deposited by Direct MOCVD

**DOI:** 10.3390/ma17010211

**Published:** 2023-12-30

**Authors:** Abderrazzak Ait Bassou, Lisete Fernandes, José R. Fernandes, Fábio G. Figueiras, Pedro B. Tavares

**Affiliations:** 1 Centro de Química-Vila Real (CQVR), Departamento Física, Escola de Ciências e Tecnologia (ECT), Universidade de Trás-os-Montes e Alto Douro, 5000-801 Vila Real, Portugal; jraf@utad.pt; 2 Centro de Química-Vila Real (CQVR), Unidade de Microscopia Eletrónica-Centro de Investigaçãoe Desenvolvimento (UME/CIDE), Universidade de Trás-os-Montes e Alto Douro, 5000-801 Vila Real, Portugal; lisfernandes@utad.pt; 3 Instituto de Física de Materiais Avançados, Nanotecnologia e Fotónica (IFIMUP), Departamento Física e Astronomia, Faculdade Ciências, Universidade Porto, 687, 4169-007 Porto, Portugal; 4 Centro de Química-Vila Real (CQVR), Departamento Química, Escola de Ciências da Vida e do Ambiente (ECVA), Universidade de Trás-os-Montes e Alto Douro, 5000-801 Vila Real, Portugal; ptavares@utad.pt

**Keywords:** oxides, thin films, MOCVD, photo-ferroelectrics, narrow band gap

## Abstract

In this work, we investigated the MOCVD conditions to synthesize thin films with the hexagonal *P6_3_cm h*-LuMnO_3_ phase as a potential low-band gap ferroelectric material. The main parameters investigated were the ratio of organometallic starting materials, substrate temperature, and annealing effect. Two different substrates were used in the study: fused silica (SiO_2_) glass and platinized silicon (Pt\Ti\SiO_2_\Si(100)). In order to investigate the thermodynamic stability and quality of the developed phases, a detailed analysis of the crystal structure, microstructure, morphology, and roughness of the films was performed by X-ray diffractometer, scanning electron microscopy (SEM), energy dispersive spectrometry (EDS), Raman spectroscopy, and piezoelectric force microscopy (PFM). Molar compositions in the film within 0.93 < |Lu|/|Mn| < 1.33 were found to be suitable for obtaining a single-phase *h*-LuMnO_3_. The best films were obtained by depositions at 700 °C, followed by thermal treatments at 800 °C for long periods of up to 12 h. These films exhibited a highly crystalline hexagonal single phase with a relatively narrow direct band gap, around 1.5 eV, which is within the expected values for the *h*-LuMnO_3_ system.

## 1. Introduction

Rare earth manganite *R*MnO_3_ are one of the most studied multiferroic materials [1,2]. Depending on the radius of the rare earth component, they usually crystallize in an orthorhombic (*o*-) structure with a *Pnma* space group for larger *R* cation radii (*R* = Ba, Bi, La, …, Dy) or in a hexagonal (*h*-) lattice with a *P6_3_cm* space group for smaller *R* cation radii (*R* = Y, Sc, In, Er, Lu). The hexagonal structures are built from MnO_5_ bipyramids that form layers separated by the *R* ions [2,3]. They exhibit an interestingly high ferroelectric (FE) transition temperature (T_C_ > 500 K) but relatively weak electric polarizations. They can also be considered multiferroic [4,5] due to antiferromagnetic (AFM) ordering at relatively low temperatures (T_N_ < 130 K), as stated in Table 1. Generally, bulk RMnO_3_ systems have been extensively studied as polycrystalline samples prepared by conventional ceramic or sol–gel methods [4] and as single crystals synthesized by floating zone methods. As thin films, they present additional degrees of freedom to be explored, such as preferential orientations, substrate-induced strain phases, and interfacial modifications, which open practical paths for the development of functional micro- and nano-devices [2]. Particular interest in the further study of *h*-*R*MnO_3_ systems stems from their relatively low-band gap (*E_g_*) combined with ferroelectric properties and their potential as photoactive materials for innovative photovoltaic and photocatalytic applications [6,7]. This is especially the case in the present time, when the development of new and efficient solar energy conversion technologies is crucial from an environmental, social, and economic point of view.

The group of Fujimura et al. were the first to report the deposition of *h*-YMnO_3_ thin films on MgO(111), ZnO(0001), Al_2_O_3_(0001) sapphire, and Pt(111)\MgO (111) using RF magnetron sputtering (RFMS) [20,21,22,23]. Subsequently, other studies were made on *h*-YMnO_3_ grown on Pt\Al_2_O_3_(0001) and on Pt\Y_2_O_3_\Si(111) structures using molecular beam epitaxy (MBE) [24]. These techniques usually require relatively expensive equipment and use bulky targets of fixed composition. On the other hand, chemical methods [4] have the advantage of comparative low cost, providing versatility in precursor composition and thus prompt control of film stoichiometry [18]. In particular, the metal organic chemical vapor deposition (MOCVD) method allows for the investigation of a series of relevant experimental parameters in order to establish suitable deposition conditions to synthetize thin films with a single hexagonal phase. Gerald et al. were able to deposit *h*-YMnO_3_, *h*-HoMnO_3_, and *h*-ErMnO_3_ on ZrO_2_(111)\(Y_2_O_3_) and on Pt(111)\TiO_2_\SiO_2_\Si(001) substrates by pulsed injection metal organic chemical vapor deposition (PIMOCVD) [25,26,27]. Their work exemplifies some physical and electrical property modifications, like magnetoelectric effects, obtained in the films by the choice of *R*MnO_3_, strain engineering via substrate, film thickness, and annealing conditions. *h*-LuMnO_3_ is one of the least studied materials in this family. The band structure published in the literature points to values of *E_g_* between 1.19 and 1.45 eV [19,28]. In their work, using DFT methods, Brito et al. calculated an indirect *E_g_* of 1.19 eV and a direct *E_g_* of 1.29 eV [28]; Souchkov et al. estimated a value of *E_g_* to be 1.1–1.5 eV on single crystals [22,29]. Han et al. described an indirect *E_g_* of 1.45 eV and a direct *E_g_* of 1.48 eV using ab initio calculations. They also measured a value of 1.555 ± 0.025 eV on thin films deposited on Pt\Al_2_O_3_ substrate, resorting to the pulsed laser deposition (PLD) technique [19].

In this study, we explored the direct MOCVD method to deposit the *h*-LuMnO_3_ phase thin films on standard fused silica glass (abbreviated to \SiO_2_) and silicon platinized substrates Pt\Ti\SiO_2_\Si(100) (abbreviated to \Pt\Si). The experimental work scrutinized the effects of the precursor ratio, deposition temperatures, atmosphere (O_2_:Ar ratio), and in situ or ex situ thermal treatments. The quality and properties of the developed films were characterized by X-ray diffraction, scanning electron microscopy (SEM), energy dispersive spectrometry (EDS), Raman spectroscopy (MRS), piezo force microscopy (PFM), and transmittance and reflectance measurements.

## 2. Experimental Procedures

The metal organic precursors of Mn(*tmhd*)_3_ and Lu(*tmhd*)_3_ were synthesized from *tmhd* (2,2,6,6-tetramethyl-3,5-heptanedione) with recourse to the method described by Eisentraut and Severs [30]. In short, Mn(NO_3_)_3_·4.H_2_O (ABCR, Karlsruhe, Germany, 8 mmol) was dissolved in water (30 mL) and slowly poured over an alkaline (0.64 g NaOH) ethanol solution (30 mL) of *tmhd* (16 mmol). Adding 250 mL of cold water produced a precipitate of Mn(*tmhd*)_3_. The precipitate was filtered and washed with cold water. In the case of Lu(*tmhd*)_3_, we used Lu(NO_3_)_3_·6.H_2_O (Alfa Aesar, Ward Hill, MA, USA) and methanol as the solvent. After vacuum drying using P_4_O_10_ as a water absorbent, the compounds were purified through sublimation. The sublimation temperatures were 110–120 °C for Mn(*tmhd*)_3_ and 140–150 °C for Lu(*tmhd*)_3_.

LuMnO_3_ thin films were deposited by the direct MOCVD method simultaneously on standard \SiO_2_ substrates and on Pt\Ti\SiO_2_\Si(100). (Radiant, Albuquerque, NM, USA) (Figure S1 in Supplementary Materials). The metalorganic precursor powders were placed in a small crucible in the sublimation zone and slowly heated from 90 °C up to 140 °C at 1 °C/min. Preheated Ar:O_2_ (2.6:179 mL/min) was used as a carrier gas. The substrates were glued using silver paste to a stainless steel susceptor heated by an induction coil (2 kW). The deposition temperature was measured using a K-type thermocouple located inside the susceptor just above the substrates and controlled by a PID Eurotherm controller (Worthing, United Kingdom). The deposition temperature was calibrated using the melting points of NaCl (801 °C) and KCl (770 °C). Typical thermodynamic deposition conditions were pressure at 9 mbar (measured with a Wenzel A200, Wiesthal, Germany) and temperature at 700 °C. The metal–organic compounds sublimated and flowed toward the substrates, where complex oxidation reactions took place. The film layer was formed, and the gaseous residues were evacuated by a pumping system through a liquid N_2_ trap. After the deposition, the films were in situ annealed for 1–12 h in different environments: pure O_2_; pure argon; or mixture at 1 bar. Ex situ annealing was conducted in a tubular oven also at 1 bar of flowing gas.

X-ray diffraction patterns were acquired using a Panalytical X’Pert Pro MPD (Almelo, The Netherlands) equipped with a X’Celerator detector and a secondary monochromator in Bragg–Brentano geometry, with λ(Cu_Kα1_) = 1.5418 Å; 2*θ* step size 0.017° at 100 s/step. The diffractograms were analyzed using PowderCell software (version 2.4). Average crystallite sizes, <*t_c_>*, were calculated using the Scherrer equation: <*t_c_>* = *K*.*λ*/*β*.*cosθ*, where *K* is a dimensionless shape factor with a typical value of 0.90, *λ* is the X-ray wavelength, *β* is the line broadening at half the maximum intensity (FWHM), after subtracting the instrumental line broadening, in radians, and *θ* is the Bragg angle.

Scanning Electron Microscopy (SEM) and Energy Dispersive Spectrometry (EDS) were performed using a FEI Quanta 400 with W filament and an EDAX system, respectively. EDS acquisition spectra were performed at 15 kV to maximize the signal from the film, and the semi-quantification was performed standardless with ZAF correction factors without considering the elements from the substrates.

Transmittance measurements were performed by UV-Vis spectroscopy using the LLG-uniSPEC 2 (Meckenheim, Germany)system to measure the light absorption behavior of the material as a function of the incident wavelength. The background was acquired using the \SiO_2_ glass substrate. Diffuse reflectance measurements were obtained using a CARY 50 Varian spectrophotometer (Agilent, Santa Clara, CA, USA) in a range from 200 to 1000 nm, using the BaSO_4_ compound as the white background reference. The acquired diffuse reflectance spectrum was converted using the Kubelka–Munk function, where the magnitude *F(R_∞_)* is proportional to the absorption coefficient. The optical band gap (*E_g_*) can be calculated following the relation presented by *Tauc* and expressed by *Davis* and *Mott*: (*α*.E)^1/*n*^ = (*h.υ* − E_g_), where E = *h.c/λ* is the photon energy and *E_g_* the optical band gap energy. The power-law exponent, *n*, depends on the transition type, using *n* = 1/2 for a direct *E_g_* and *n* = 2 for an indirect *E_g_*. The value of indirect or direct E_g_ is estimated by plotting (*α.h.υ*)^1/2^ or (*α*.*h.υ*)^2^, respectively, as a function of the photon energy and extrapolating it to 0 [19,31]. In this work, the first derivative method [32] was also used in complement to Tauc’s plot method.

Piezo-response force microscopy was performed using a scanning probe microscope NT-MDT NTEGRA equipped with an external lock-in amplifier Zurich instruments HFLI (Zürich, Switzerland). Commercial probes from Budget Sensors Tap190E-G (Sofia, Bulgaria) were used, with Cr/Pt-coated tips of a radius of 25 nm, a resonance frequency of ~151 kHz, and a spring constant *k* of about 48 N/m. All piezo-response force microscopy and spectroscopy studies were performed outside the 21.1(1) kHz resonance frequency in order to decrease electrostatic responses and topographic crosstalk.^23^ The images were edited with the WSxM 5.0-10.0 software.

## 3. Results and Discussion

### 3.1. Film Composition and Phases

**Figure 1:**
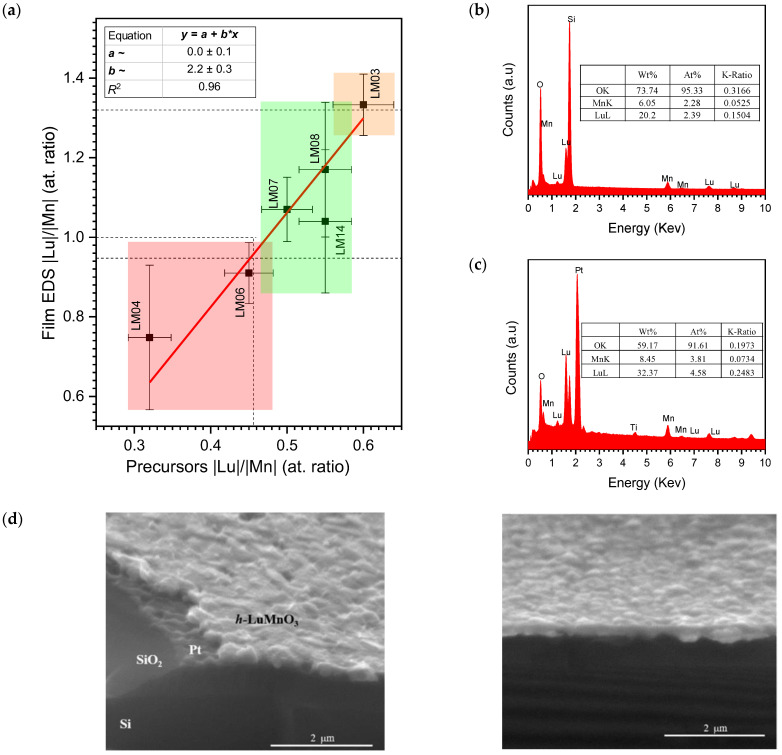
(**a**) Atomic ratios Lu/Mn used in the precursors and resulting films. Representative EDS spectra of film LM800-*in*12\Pt\Si: (**b**) \SiO_2_ glass, and (**c**) on \Pt\Si substrate. (**d**) Representative cross-section images of film LM800-*in*12\Pt\Si.

The first series of experimental depositions was used to calibrate the transfer function of the precursor molar ratio to the resulting film composition. Figure 1a) shows the experimental data obtained for different |Lu(*tmhd*)_3_|/|Mn(*tmhd*)_3_| ratios from 0.30 to 0.60. Under the specifics of the reactor and deposition conditions used, a simple linear fit allowed interpolation of the value |Lu(*tmhd*)_3_|/|Mn(*tmhd*)_3_| = 0.47 ± 0.03 as an indicator of stoichiometry approaching |Lu|/|Mn| = 1.00 in the films, as measured by EDS\SEM. The rate of sublimation of Lu(*tmhd*)_3_ and incorporation of Lu cations into the film phase are relatively more effective than that of Mn(*tmhd*)_3_ and Mn. Representative EDS spectra of the Lu-Mn-O thin films are shown in Figure 1b,c), which confirm the presence of the expected elements with no other impurities detected. However, quantification of the Lu and Mn ratios in the film by the EDS method can be determined only within a relatively significant margin of error because the electron beam penetrates successive layers in addition to the thin film and is perturbed by each interface [33], and it also penetrates into the fused silica or the Pt, Ti, and SiO_2_ buffers and reaching the Si substrate. Representative cross-section images are presented in Figure 1d and enable us to estimate the film thickness at around 230 ± 20 nm.

**Figure 2:**
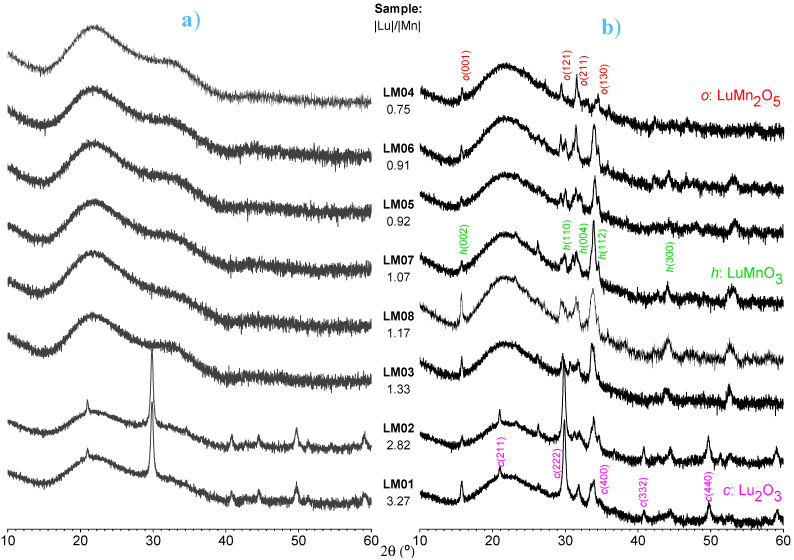
XRD pattern of Lu-Mn-O films deposited on \SiO_2_; (**a**) *as-deposited* at 700 °C, (**b**) after ex situ anneal at 800 °C 1 h.

Figure 2 compares the XRD patterns of a series of layers deposited at 700 °C on \SiO_2_ from different feedstocks. The layers are also labeled with the corresponding Lu/Mn ratios determined using the EDS technique. The three color-shaded regions in Figure 1a group the different phases identified in the thin films in correlation to their respective compositions. Regardless of the ratio of the starting materials, the XRD patterns of the films shown in Figure 2a were generally amorphous in their initial state. The XRD patterns shown in Figure 2b were obtained after the samples were subjected to ex situ heat treatment at 800 °C for 1 h in air flow at atmospheric pressure. As can be seen, annealing promoted crystallization in all films. However, different structural phases were formed depending on the Lu/Mn ratio in each film. Under the specifications of the experimental MOCVD system, deposits with an excess of the Lu(*tmhd*)_3_ precursor resulted in films with |Lu|/|Mn| > 1.33 and developed t *h*-LuMnO_3_ (Crystallography Open Database card n. 9007909) and cubic *c*-Lu_2_O_3_ (Crystallography Open Database card n. 1548519) phases. Deposits with a deficit of Lu(*tmhd*)_3_, on the other hand, produced films with |Lu|/|Mn| < 0.92 and exhibited a mixture of *h*-LuMnO_3_ and orthorhombic o-LuM_2_nO_5_ phases. For films within a composition window of 0.93 < |Lu|/|Mn| < 1.33, which corresponds to an excess of Mn(*tmhd*)_3_ (up to a maximum ratio of 0.55 in the precursor solution), it appears to be possible to synthesize a thin film with a single *h*-LuMnO_3_ *P6_3_cm* phase. In addition to optimizing appropriate thermal treatments, the XRD proves that it is possible to obtain the *h*-LuMnO_3_ phase in a relatively wide range of Lu/Mn ratios.

### 3.2. Thermal Treatments

Since the compositional parameters used for the synthesis of sample LM08 were found to be suitable for producing a single-phase *h*-LuMnO_3_ film on the \SiO_2_ substrates, a series of films were subsequently deposited simultaneously on the \SiO_2_ and on \Pt\Si substrates, with the precursor ratio fixed at |Lu|/|Mn| ~0.55. Further experiments were conducted to investigate the effects of in situ and ex situ heat treatments on the evolution of the phase. One series was deposited at 700 °C (LM700-*as*00) and then annealed ex situ at 800 °C in air for 12 h (LM700-*ex*12). Other series were deposited at 800 °C (LM800-*as*00) and further annealed ex situ at 800 °C in air for 12 h (LM800-*ex*12). Other films deposited at 800 °C were annealed in situ for 4 h (LM800-*in*04) and 12 h (LM800-*in*12), as shown in Table 2.

**Figure 3:**
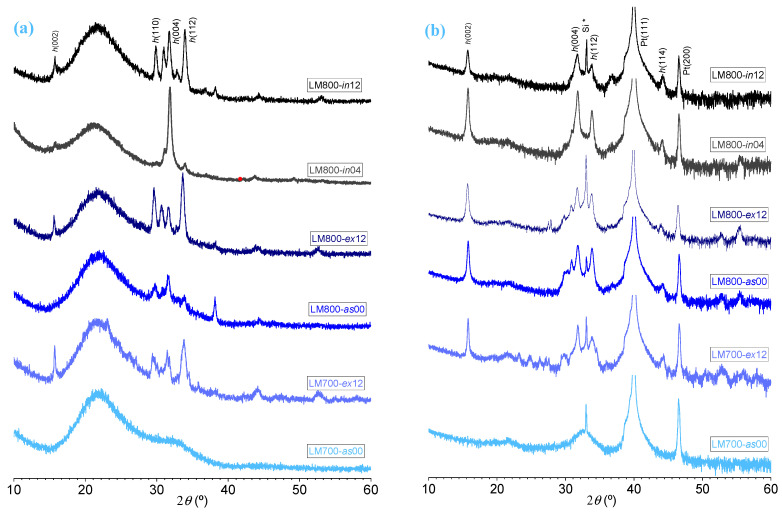
XRD patterns of film series deposited on (**a**) SiO_2_ glass and (**b**) Pt\Si substrates with different thermal treatments. The Si * is related to the “forbidden” Si(200) plane reflection.

Figure 3a,b, respectively, show both the XRD patterns of the film series deposited on the SiO_2_-glass and Pt\Si substrates and the microscopic surface images obtained using SEM. As shown in Figure 3a, the LM700-*as*00\SiO_2_ deposited film is essentially amorphous, as corroborated by the absence of diffraction peaks. After ex situ heat treatment at 800 °C for a period of 12 h, it is possible to observe the appearance of reflection peaks (002), (004), and (112) of *h*-LuMnO_3_ in the diffractogram of film LM700-*ex*12\SiO_2_. These are related to the crystallization of the hexagonal phase and allow for the calculation of the cell parameters *a* = 6.002(2) Å and *c* = 11.27(2) Å, as displayed in Table 2. On the other hand, when the film is directly deposited at 800 °C, the XRD of sample LM800-*as*00\SiO_2_ shows already the (110), (004), and (112) diffraction peaks, albeit with low intensity, revealing incipient crystallization (crystallite size is 16 ± 2 nm). The cell volume is 351(1) Å^3^, which is 2.2% smaller than the bulk volume for a stoichiometric sample [34]. After ex situ annealing at 800 °C for 12 h in air (LM800-*ex*12\SiO_2_), the crystallization increases, the (002) peak appears, and the other reflection peaks become much more intense with a smaller FWHM, indicating the growth of the crystallites (20 ± 1 nm). In addition, the lattice and cell volume increase toward bulk values. The diffractogram of the LM800-*in*04\SiO_2_ film deposited at 800 °C and annealed in situ for 4 h in oxygen indicates preferentially oriented growth along the *c*-axis of the crystallized phase. A major reflection peak at 31.85° (004) and a minor peak at 34.00° (112) allow the calculation of the lattice parameters *a* = 5.97(2) Å and *c* = 11.22(2) Å, with a shrinkage of the cell volume to 346(2) Å^3^. Almost the same shrinkage is observed in the LM800-*in*12\SiO_2_ film, annealed in situ for 12 h in oxygen.

Figure 3b shows the XRD pattern of the films deposited on the \Pt\Si substrates under the same batch as the films deposited on SiO_2_ glass. The narrow diffraction peak located at 2θ = 33.0° is related to the “forbidden” Si(200) plane reflection [35]. The platinum peaks appear at 39.95° (111) and 46.50° (200). The phase formation is not relevant for the LM700-*as*00\Pt\Si sample. Then, the crystallization of the films subjected to ex situ annealing reveals an improvement. In the LM700-ex12\Pt\Si film, the crystallite size approaches 25 nm approximately, while in the LM800-ex12\Pt\Si film, the crystallite size becomes more noticeable, reaching around 31 nm. This is particularly evident from the reflection peaks of the (002), (004), and (112) planes of the hexagonal structure. The X-ray diffractograms of the in situ annealed films (LM800-*in*04\Pt\Si and LM800-*in*12\Pt\Si) have nearly the same lattice parameters, exhibiting cell volumes of 350(1) Å^3^ and 349(2) Å^3^.

In general, the lattice parameters of the films deposited on Pt\Si show minor variations with the thermal treatments. These differences are consistent with a stronger phase adhesion induced by the crystalline template, in contrast to the lessen strain from the amorphous \SiO_2_ glass substrate. Moreover, prolonging the thermal treatments clearly leads to recrystallization and strain relaxation, increasing the lattice volume and improving the overall quality of the film.

**Figure 4:**
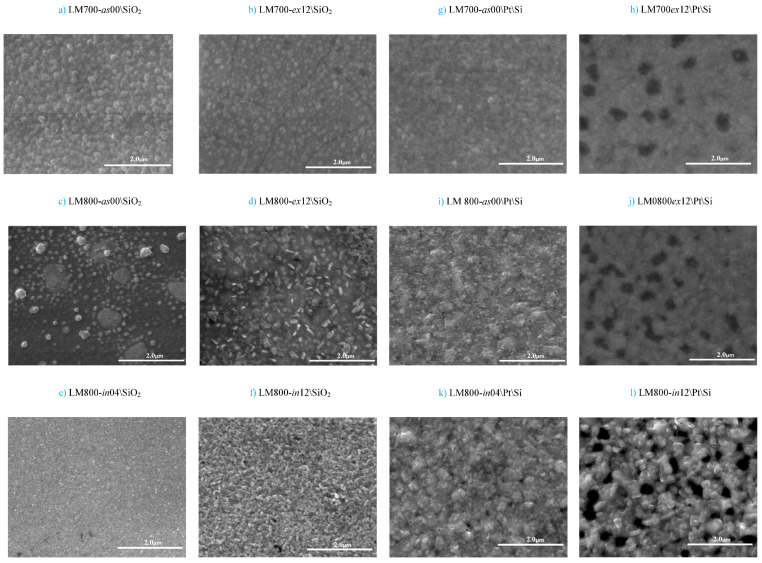
SEM images of film surface deposited on (**a**–**f**) \SiO_2_ and on \Pt\Si. (**g**–**l**) at different temperatures and thermal treatments as described in Table 2.

In agreement with the XRD observations, Figure 4 shows surface microstructure images obtained by SEM from these series of films deposited on the \SiO_2_ glass and \Pt\Si substrates. Figure 4a,g correspond to samples LM700-*as*00, which exhibit a smooth texture as expected from basically amorphous films. Relatively uniformly sized grains with a diameter of about 200 nm can be seen on the film on SiO_2_ glass (Figure 4a), while they are barely visible in the \Pt\Si films. After annealing at 800 °C for 12 h, the LM700-*ex*12 film (Figure 4b) developed some crystallization as well as a structural contraction of the original amorphous phase, which resulted in a denser surface and an abundance of cracks. This transformation is evident in the partial growth of some grains at the expense of neighboring regions, which turned the previously uniform texture into an irregular distribution of crystallite sizes, reaching up to 300 nm in diameter. By contrast, the LM800-*in*04 film (Figure 4e) has a very dense, homogeneous, and smooth surface; it exhibits a highly dispersed nucleation and a crystallite below 20 nm, but no aggregates or visible contours. The deposited films at 800 °C on/SiO_2_ substrate (Figure 4c) shows inhomogeneous growth, which leads to the appearance of some outgrowth on the surface of the films. we assume that the film does not have enough time to crystallize well even though at high temperature. However, after TT at 800 °C for 12 h, the film (Figure 4d) shows a better crystallization with very small grains and develops some cracks due to thermal expansion. The LM800-*in*12 film (Figure 4f) shows a recrystallized surface with a uniform distribution of grains with a diameter of about 175 nm but no signs of aggregates or cracks. In contrast with the relatively smooth and incipient microstructure observed for the films deposited on \SiO_2_ glass, those deposited on \Pt\Si show some relevant differences. With a limited annealing time of 4 h, the film develops a very dense and crystallized surface with crystallites of less than 200 nm in size and very dispersed porosity. However, after a 12 h annealing, (Figure 4h,j,l), the films develop significant recrystallization, although they are affected by some degree of porosity that can reach 250–350 nm-wide voids, possibly reaching the substrate surface (a SEM image of \Pt\Si substrate is given in Figure S2 Supplementary Materials). These results indicate that both the substrate and the thermal treatment have profound effects on the evolution of the film phase microstructure and may eventually further affect its physical properties.

In summary, the XRD diffractograms show a relative increase in the intensity and definition of the reflection peaks of the *P6_3_cm* phase, indicating an improvement in crystallization quality with the longer in situ annealing time. The images from SEM show the importance of choosing a higher deposition temperature to improve the film morphology. Thus, the combination of deposition and sufficiently long thermal treatments promotes not only the *h*-LuMnO_3_ phase crystallization as a denser film. The optimized films exhibit the intended hexagonal *P6_3_cm* phase, with the lattice parameters approaching those previously reported for ceramics [34,36] and single crystals [37]. As with films deposited by other methods such as spin coating [38], a relative contraction of the cell volume of the films is observed, between −0.39 and −3.3% compared to the bulk [34,36], mainly due to the strain induced by the substrates, besides slightly off-stoichiometry effects [39]. If one expresses the composition of the hexagonal phase as LuMn_1−*z*_O_3−*δ*_ [34], it is evident that both the Lu/Mn composition (imposing *z*) and the air, the O_2_/Ar atmosphere (affecting *δ*), are two important chemical factors that impact the lattice volume [39]. Nonetheless, the changes observed in the lattice parameters due to thermal treatments should also be considered influenced by the degree of film strain imposed from the substrates, or conversely, the degree of relaxation due to recrystallization.

### 3.3. Raman Spectroscopy

**Figure 5:**
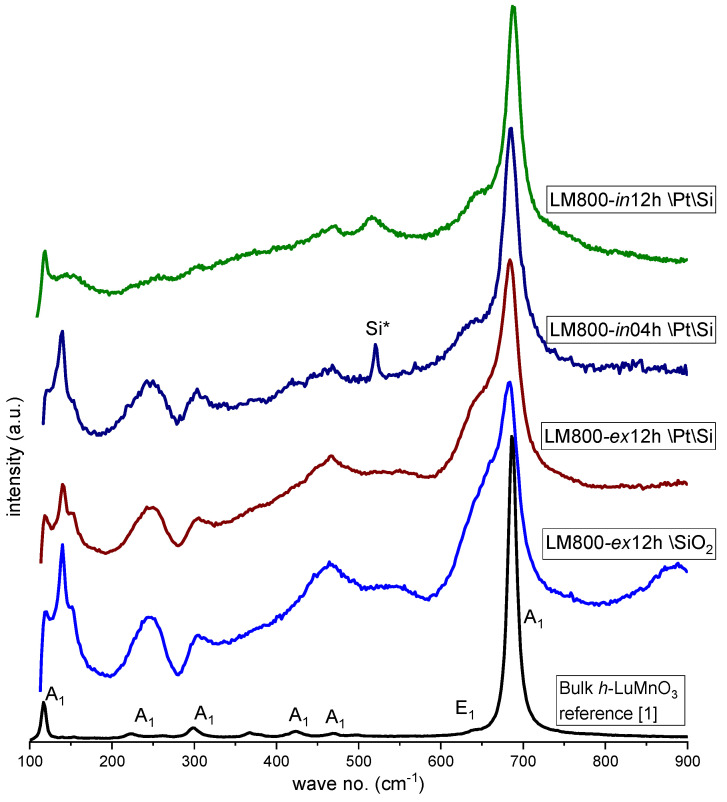
Raman spectra of *h*-LuMnO_3_ films prepared by CVD and bulk reference [1]. Si* represents the Si substrate peak.

The quality of the *h*-LuMnO_3_ phase in the films was further inspected using Raman spectroscopy. The spectra obtained from the different films also allowed us to follow the effects of adjustments in the deposition conditions and thermal treatments. The characteristic Raman profile of the *h*-LuMO_3_ *P6_3_cm* phase has 38 Raman active phonon modes: 9 A_1_,14 E_1,_ and 15 E_2_ [40,41]. The series of films deposited on SiO_2_ glass were mainly used to parametrize the composition. The results of XRD and the analysis of SEM images indicate limited crystallization, and corresponding Raman spectra confirm the incipient formation of the hexagonal phase. Subsequent series of films deposited on the platinized substrates show an improvement in the quality of hexagonal phase crystallization, and Figure 5 shows some representative Raman spectra from the film at ambient conditions. Apart from the peak at 516 cm^−1^ from the Si substrate [42], the spectra approach the phonon modes of the *P6_3_cm* phase, in particular the series of A_1_ modes (around 118, 224, 301, 472, and 689 cm^−1^) and E_1_ (around 642 cm^−1^) as reported in the literature [40] (Table 3).

Even though the films are dominated by the hexagonal crystalline phase, the corresponding Raman spectra are clearly distorted from those of the typical *h*-LuMO_3_ bulk structure. The position of the main A_1_ phonon modes appears to be slightly shifted to the lower wave number (~685 cm^−1^), as expected due to the film strain and lattice contraction. In addition, broader peaks and shoulders are typical of significant lattice distortions as well as disorder and defects in the film [28]. Furthermore, distortions at interface regions and the possible superposition of modes from spurious phases, though not detected by XRD, typically contribute to convolutions and perturbations in the Raman spectra.

### 3.4. Optical Band Gap Measurements

**Figure 6:**
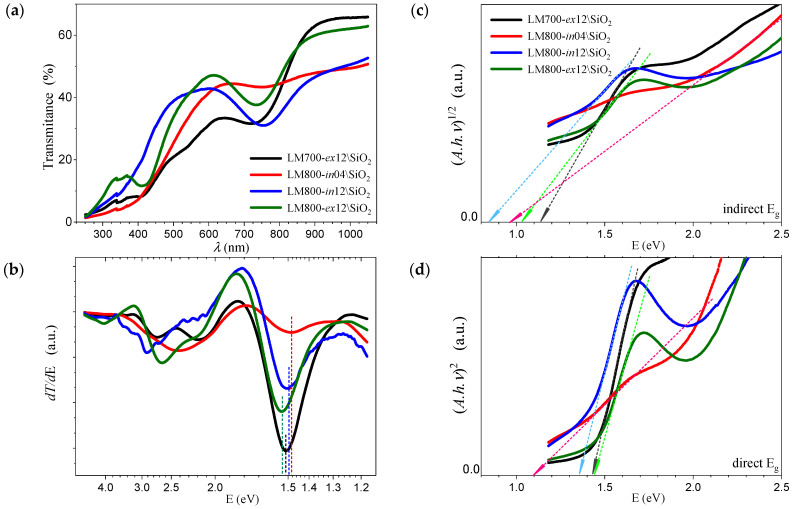
*h*-LuMnO_3_ films on SiO_2_ substrates (**a**) transmittance and its (**b**) derivative; calculated (**c**) indirect, and (**d**) direct *E_g_*.

**Figure 7:**
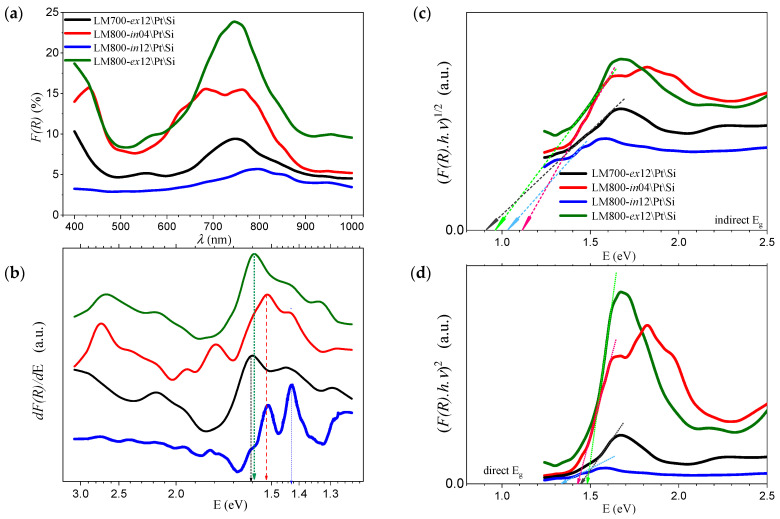
*h*-LuMnO_3_ films on \Pt\Si substrates (**a**) reflectance and its (**b**) derivative; calculated (**c**) indirect and (**d**) direct *E_g_*.

Figure 6a shows the change in transmittance as a function of incident wavelength for the coatings deposited on \SiO_2_ at different annealing conditions. The infrared transmittance can reach 50 to 65% above 900 nm, while a significant decrease to almost 40% is observed below 800 nm. The derivative of transmittance (*dT/dE*) shown in Figure 6b shows a uniform absorption threshold for photons with an energy of about 1.5 eV for all samples. This behavior is clearly enhanced for the three samples with a longer 12 h thermal treatment, while it is only at the beginning for the sample with a 4 h annealing [43]. The corresponding *Tauc* plots are shown in Figure 6c,d and allow the calculation and verification of more unambiguous values of optical band gap (*E_g_*), which are reproduced in Table 4. It is possible to extrapolate values for indirect *E_g_* within 0.9 to 1.1 eV, although this is not apparent for the LM800-*in*04 film, which shows an absorption near 1.4 eV. In addition, the direct *E_g_* values are estimated to be between 1.35 for the LM800-*in*12 film and a maximum of 1.68 eV for the LM800-*in*04 film. Figure 7a–d show the results of reflectance measurements of the films deposited on \Pt\Si substrates. The successive local maxima observed in the derivative (*dF(R)*/*dE*) can be associated with the distribution of density of states in the material, with differentiated gaps and efficiencies for electron transference from valence band to conduction band across the reciprocal lattice directions [28]. The calculated values of *E_g_* are listed in Table 4. The indirect *E_g_* values range from 0.9 to 1.1 eV, and the direct *E_g_* values range from 1.3 to 1.5 eV and are very similar to the values for the samples prepared on the \SiO_2_ glass substrates.

The independent values obtained for the *h*-LuMnO_3_ films on the different substrates and by different methods are close to 1 eV, within a consistent and relatively narrow interval, and in close agreement with the values reported for other *h*-LuMnO_3_ thin films [19,22], which are here reported for the first time in thin films made by MOCVD and deposited on the \SiO_2_ substrates.

### 3.5. Piezo Force Microscopy Measurements

**Figure 8:**
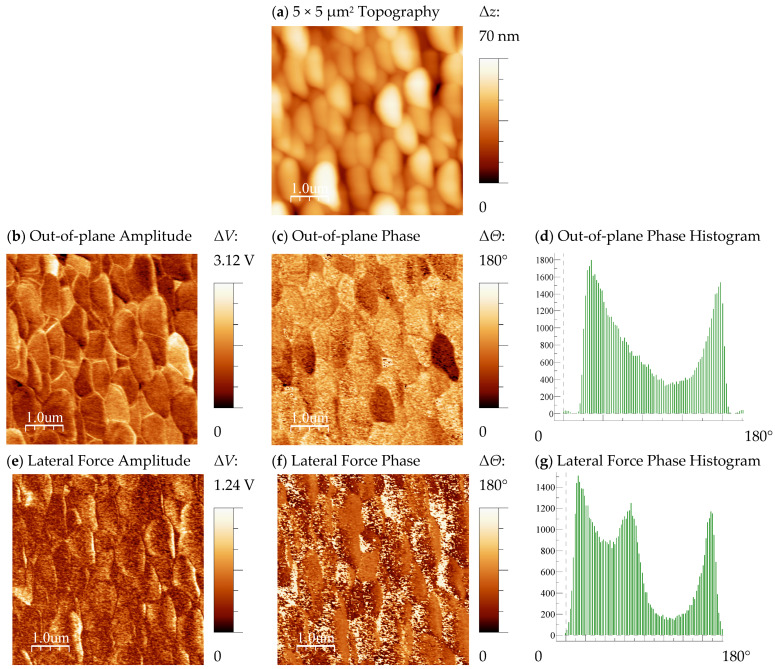
PFM scan of the surface of a LM800-*in*12 film grown on \Pt\Si through a top Au electrode thin film.

Standard arrays of Au electrode dots (1 mm^2^) were deposited with recourse to dc-sputtering in order to characterize the films’ transport and polarization properties. Due to their porosity, these Au electrodes tend to form a short circuit with the Pt substrate layer, which makes them unsuitable for conventional macroscopic dielectric or polarization measurements. Nevertheless, local microscopic evaluation of the piezoelectric response of the deposited layer is possible using the PFM technique. Figure 8a–g show PFM scans and histograms of the LM800-*in*12\Pt\Si film’s surface, measured from the top deposited Au layer. The top conductive electrode is intended to neutralize artifacts from electrostatic charge accumulation. In fact, overall similar PFM results were checked directly on the film phase surface since the film exhibits relatively low resistivity. Figure 8a displays the topography of the scanned 5 × 5 µm^2^ area. The surface of the film exhibits an average roughness below 7 nm; it consists of a dense packing of regular-size crystallites close to 1 µm wide. The Au layer was found to mimic the morphology of the underlying oxide film. Moreover, the quality of the piezo-response signal-to-noise ratio is enhanced using the Au coating in comparison to the scans performed on the uncoated film surface. The out-of-plane piezo-response amplitude and phase scans observed in Figure 8b,c observe some crosstalk to the topography, even when measured through the top Au electrode. The contrast distribution alongside grain boundaries suggests that these can also act as pinned domain walls. The respective phase histogram, depicted in Figure 8d, is distributed by two main peaks with a near 180° offset, which is indicative of a preferential orientation of the piezo-response domains (or domain walls) aligned in an out-of-plane direction with the external electric field. Further confirmation of the presence of domains can be observed from the lateral force (in-plane) piezo-response amplitude and phase scans seen in Figure 8e,f. The in-plane contrasting regions encompass several grains, and the histogram displayed in Figure 8g shows a distribution by three main peaks. Hence, this result proofs that a significant part of the domains and domain walls have a distribution of piezo-response orientation around the out-of-plane. Equivalent experiments were performed on a LM800-*in*04 sample. The results obtained were similar and confirm both the presence of piezoelectric properties and a typical ferroelectric-like domain structure.

## 4. Conclusions

In this study, we deposited the *h*-LuMnO_3_ thin films on fused silica glass and platinized silicon substrates using the MOCVD technique. The results show that the formation of the *h*-LuMnO_3_ phase mainly depends on the molar ratio of the precursor (Lu/Mn) and the deposition temperature. It was found that the *h*-LuMnO_3_ phase formation can be achieved within 0.93 < |Lu|/|Mn| < 1.33. Moreover, the films deposited at 700 °C were all amorphous, regardless of the ratio of the starting materials used. A 1 h thermal treatment at 800 °C in air was sufficient to initiate crystallization and produce different phases, which strongly depended on the ratio of the starting materials. The XRD and Raman results indicate critical effects of thermal treatment on the quality of the growth phase. The in situ and ex situ heat treatments for 12 h produced a well-crystallized phase. The films grown on amorphous fused silica glass allowed the direct measurement of transmittance and facilitated the band gap measurement using the first derivative and *Tauc* models. The results showed a low-band gap between the films, with values around 1 eV for indirect *E_g_* and 1.5 eV for direct *E_g_*. These values confirm the theoretical calculations in the literature and are considered very suitable values for applications in photovoltaics and photoactive materials. The results of the current work are very promising and show the ability of the direct MOCVD to produce a well crystalized films on various substrates. However, the presence of the porosity on the \Pt\Si substrates and films deposited hampered further electrical measurements. Further studies are expected for a higher quality substrates and should be expanded to investigate the ferroelectric measurements and bulk photovoltaic effect, because the produced films show the necessary low-band gap feature for solar energy harvesting.

## Figures and Tables

**Table 1:** Lattice parameters, Curie temperatures, and magnetic ordering temperatures of hexagonal RMnO_3_.

RMnO_3_	*a* (Å)	*c* (Å)	*T*_N_ (K)	*T*_c_ (K)	*E_g_* (eV)	References
InMnO_3_	5.869	11.47	120	500	1.16	[6,7,8]
ScMnO_3_	5.833	11.17	130	--	--	[7]
YMnO_3_	6.148	11.44	72	920	1.53–2.10	[7,9,10,11]
DyMnO_3_	6.182	11.45	39–57	19	--	[7,9,12,13]
HoMnO_3_	6.142	11.42	76	873	1.35–1.40	[7,9,14,15]
ErMnO_3_	6.112	11.40	79–81	800	1.35	[7,15,16]
TmMnO_3_	6.092	11.37	84–86	>573	--	[7,9]
YbMnO_3_	6.062	11.36	87–89	993	1.35	[7,9,15]
TbMnO_3_	6.270	11.46	41–42	>590	1.4	[17,18]
LuMnO_3_	6.046	11.41	90	>750	1.55	[7,9,19]

**Table 2:** Deposition and annealing conditions and lattice parameters for the series of films on \SiO_2_ and on \Pt\Si substrates.

Sample	Substrates	Deposition Temperature	Thermal Treatment	Time (h)	*a* (Å)	*c* (Å)	V (Å^3^)	ΔV/V_bulk_ [34] (%)
LM700-*as*00	\SiO_2_ \Pt\Si	700 °C	*as-deposited*	00	-- --	-- --	-- --	-- --
LM700-*ex*12	\SiO_2_ \Pt\Si		ex situ 800 °C 1 bar air	12	6.002(2) 5.99(3)	11.27(2) 11.23(2)	351(1) 349(3)	−2.2 −2.9
LM800-*as*00	\SiO_2_ \Pt\Si	800 °C	*as-deposited*	00	5.99(3) 5.998(7)	11.28(9) 11.22(2)	350(2) 350(1)	−2.4 −2.7
LM800-*ex*12	\SiO_2_ \Pt\Si		ex situ 800 °C 1 bar air	12	6.03(2) 6.000(4)	11.34(3) 11.27(4)	357(2) 351(1)	−0.7 −2.2
LM800-*in*04	\SiO_2_ \Pt\Si		in situ 800 °C 1 bar O_2_	04	5.97(2) 5.994(3)	11.22(2) 11.24(2)	346(2) 350(1)	−3.6 −2.6
LM800-*in*12	\SiO_2_ \Pt\Si		in situ 800 °C 1 bar O_2_	12	5.98(2) 6.00(1)	11.23(4) 11.22(2)	348(2) 349(2)	−3.1 −2.7

**Table 3:** A_1_ and E_1_ Raman active modes wave no. (cm^−1^) of *h*-LuMnO_3_ samples.

Raman Mode	A_1_	--	A_1_	A_1_	A_1_	A_1_	A_1_	E_1_
Single crystal [42]	121	--	224	301	432	472	689	642
Bulk LuMnO_3_ [1]	117	--	222	298	425	463	689	640
LM800-*ex*12\SiO_2_	118	140	244	303	--	463	685	661
LM800-*ex*12\Pt\Si	116	140	247	302	--	465	684	655
LM800-*in*04\Pt\Si	118	139	241	302	--	465	684	644
LM800-*in*12\Pt\Si	118	--	--	--	--	469	688	648

**Table 4:** *E_g_* calculated by *Tauc* model for the series of *h*-LuMnO_3_ films deposited on \SiO_2_ and \Pt\Si.

Sample	Substrates	Indirect *E_g_* ±0.05 [eV]	Direct *E_g_* ±0.05 [eV]
LM700-*ex*12	\SiO_2_ \Pt\Si	1.14 0.90	1.44 1.44
LM800-*ex*12	\SiO_2_ \Pt\Si	1.04 0.96	1.44 1.48
LM800-*in*04	\SiO_2_ \Pt\Si	0.96 1.11	1.1 1.43
LM800-*in*12	\SiO_2_ \Pt\Si	0.86 1.04	1.35 1.34

## Data Availability

Data are contained within the article and Supplementary Materials.

## Supplementary Materials

The following supporting information can be downloaded at: https://www.mdpi.com/article/10.3390/ma17010211/s1, Figure S1: Diagram of the experimental apparatus used for direct metal organic chemical vapor deposition (MOCVD); Figure S2: Example of SEM image of a plain substrate of Pt\Ti\SiO_2_\Si(100) surface after thermal treatment at 800 °C in air. It is observed the formation of pores with an average size 160 ± 10 nm, independently of any film deposition.
