# Supplementary material for: Growth and Structural Characterization of h-LuMnO3 Thin Films Deposited by Direct MOCVD
Source: Materials (Basel). 2023 Dec 30;17(1):211. doi: 10.3390/ma17010211 (PMC10780143; doi:10.3390/ma17010211)
Supplement: Supplementary file 1 [file materials-17-00211-s001.zip › materials-2770564-supplementary.pdf]

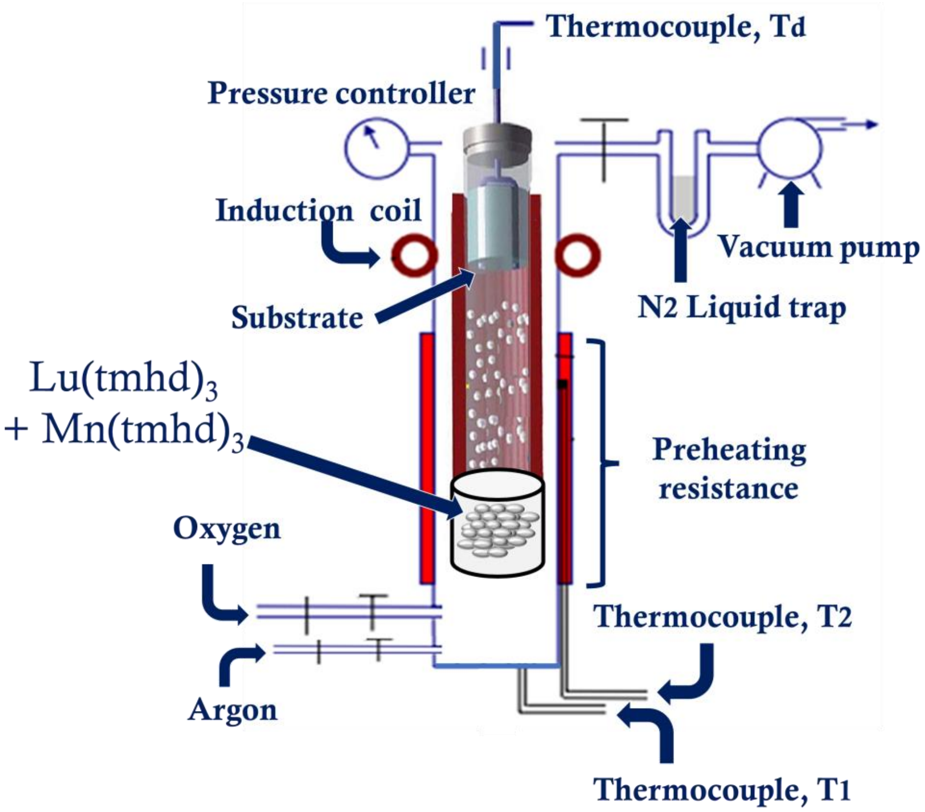

Figure S1: Diagram of the experimental apparatus used for direct metal organic chemical vapor deposition (MOCVD)

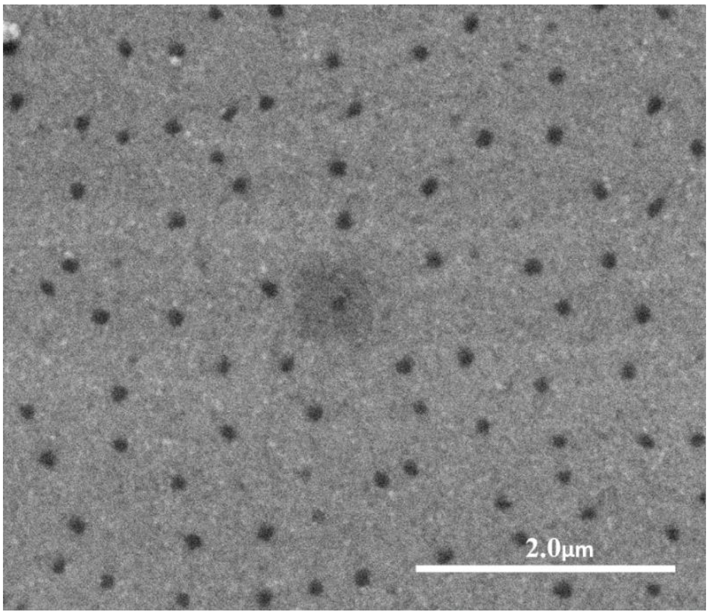

Figure S2: Example of SEM image of a plain substrate of Pt\Ti\SiO_2\Si(100*)* surface after thermal treatment at 800 °C in air. It is observed the formation of pores with an average size 160 ±10 nm, independently of any film deposition.
